# Grain Legume Yield Responses to Rhizobia Inoculants and Phosphorus Supplementation Under Ghana Soils: A Meta-Synthesis

**DOI:** 10.3389/fpls.2022.877433

**Published:** 2022-06-23

**Authors:** Alfred Balenor Buernor, Muhammad Rabiu Kabiru, Noura Bechtaoui, Jibrin Mohammed Jibrin, Michael Asante, Anis Bouraqqadi, Sara Dahhani, Yedir Ouhdouch, Mohamed Hafidi, Martin Jemo

**Affiliations:** ^1^AgroBioscience Program, University Mohammed VI Polytechnic (UM6P), Benguerir, Morocco; ^2^Centre for Dryland Agriculture, Bayero University, Kano, Nigeria; ^3^Council for Scientific and Industrial Research-Savanna Agricultural Research Institute (SARI), Tamale, Ghana; ^4^OCP Africa, Casablanca, Morocco; ^5^Laboratory of Microbial Biotechnologies, Agrosciences and Environment, Faculty of Science Semlalia, Cadi Ayyad University, Marrakesh, Morocco

**Keywords:** Ghana soils, inoculant formulation method, inoculation responses, meta-analysis, legumes, phosphorus application, rhizobium strains, yield

## Abstract

A discrete number of studies have been conducted on the effects of rhizobia (Rhz) inoculants, phosphorus (P) management, and combined application of Rhz and P fertilizer on the enhancement of grain legume yield across soils of Ghana and elsewhere. However, the extent to which the various inoculated Rhz strains, P application, and combined application of Rhz + P studies contribute to improving yield, performed on a comprehensive analysis approach, and profit farmers are yet to be understood. This study reviewed different experimental studies conducted on soybean (*Glycine max* (L.) Merr.), cowpea (*Vigna unguiculata* [L.] Walp), and groundnut (*Arachis hypogaea* [L.]) to which Rhz inoculants, P supplements, or Rhz + P combination were applied to improve the yield in Ghana. Multiple-step search combinations of published articles and multivariate analysis computing approaches were used to assess the effects of Rhz inoculation, P application, or both application of Rhz and P on yield variation. The random forest (RF) regression model was further employed to quantify the relative importance of various predictor variables on yield. The meta-analysis results showed that cowpea exhibited the highest (61.7%) and groundnut (19.8%) the lowest average yield change. The RF regression model revealed that the combined application of Rhz and P fertilizer (10.5%) and Rhz inoculation alone (7.8%) were the highest explanatory variables to predict yield variation in soybean. The Rhz + P combination, Rhz inoculation, and genotype wang-Kae explained 11.6, 10.02, and 8.04% of yield variability for cowpea, respectively. The yield in the inoculated plants increased by 1.48-, 1.26-, and 1.16-fold when compared to that in the non-inoculated cowpea plants following inoculation with BR 3299, KNUST 1002, and KNUST 1006 strains, respectively. KNUST 1006 strain exhibited the highest yield increase ratio (1.3-fold) in groundnut plants. Inoculants formulation with a viable concentration of 10^9^ cells g^−1^ and a minimum inoculum rate of 1.0 × 10^6^ cells seed^−1^ achieved the highest average yield change for soybean but not for cowpea and groundnut. The meta-analysis calls for prospective studies to investigate the minimum rate of bacterial cells required for optimum inoculation responses in cowpea and groundnut.

## Introduction

Nitrogen (N) is the macro-nutrient that is required in the largest amount for plant growth, development, and yield (Good, 2018). Its deficiency in the soils adversely impacts the overall agricultural productivity. A quick method to supply N to plants is *via* synthetic industrial production of reactive N-fertilizers to maintain higher crop yields in highly agricultural intensification systems (Allen, 2008; Good, 2018). However, in low-input production systems, such as in many smallholder farms in Africa, food production is hampered by the use of little or no N (Masso et al., 2017; Bindraban et al., 2020). Other impediments to high crop yields in sub-Saharan Africa (SSA) are the inherent low N status of soils, a high nutrient depletion rate (which is exacerbated by crop removals), and non-replenishment (Manyong et al., 2001; Jemo et al., 2015; Bado and Bationo, 2018). Increasing events of drought associated with uncertainties in climate change are also observed in many areas of West African regions, which will also likely aggravate the yield gaps, specifically for legume crops that are often grown toward the end of the rainy season (Cernay et al., 2016; van Loon et al., 2018; Defrance et al., 2020; Paliwal et al., 2020). Therefore, exploration of alternatives to synthetic N sources and fertilization, such as the natural biological N_2_ fixation (BNF), is paramount to increasing crop yields in smallholder farms, particularly in Ghana.

Unlike most non-legumes, legume crops establish a beneficial root association with bacteria in the α- and ß-proteobacteria groups, where rhizobia (Rhz) convert abundant N_2_ present in the atmosphere into usable N *via* specialized plant structures called “nodules” (Udvardi and Poole, 2013; Basu and Kumar, 2020). Through this process, legumes satisfy their N demand and that of other non-legume plants when grown in rotation or association. This process contributes to the agricultural sustainability of production systems for the benefit of millions of smallholder farmers (Giller, 2001; Desbrosses and Stougaard, 2011; Franke et al., 2018; Ulzen et al., 2018; Adjei-Nsiah et al., 2019). However, various abiotic and biotic stress factors, such as drought (Jemo et al., 2017), low phosphorus (P) availability (Sulieman and Tran, 2015), non-competitive Rhz strains, and diseases (Jiménez-Guerrero et al., 2021), may affect the BNF process and limit legume productivity (van Loon et al., 2018).

Food legumes are extensively grown in Africa, and ~100 million hectares of land are under grain legume cultivation annually (Cernay et al., 2016; van Loon et al., 2018). Grain legumes, principally soybean [*Glycine max* (L.) Merr.], cowpea (*Vigna unguiculata* [L.] Walp), and groundnut (*Arachis hypogaea* [L.]) are major sources of rich protein for human consumption and animal feed for the millions of smallholdings, and play a significant role in food security (Cernay et al., 2016; Foyer et al., 2019; Daryanto et al., 2020). Grain legume species exhibit differences in responses to rhizobia inoculation and the symbiotic N_2_ fixation process (Santachiara et al., 2017). During the last three decades, the bulk of research on the BNF processes of grain legumes across SSA has been in soybean (van Heerwaarden et al., 2018), owing to the advanced breeding programs for the development of “promiscuous nodulation” varieties (Parr et al., 2017; Khojely et al., 2018) and the development of soybean genotypes/varieties with high affinity to nodulate a specific type of bacteria (*Bradyrhizobium japonicum*) to mimic native “rhizobia competition problems” (Tang et al., 2016; Sugawara et al., 2018). Recently, new economic opportunities through the development of soybean-based supply chains and the success story of soybean in Latin America have further served as drivers for high interest in soybean cultivation (Goldsmith, 2008; Foyer et al., 2019). Other grain legumes, in particular, cowpea and groundnut, which played roles in subsistence and system maintenance of the smallholdings, only received little research interest regarding the improvement of symbiotic effectiveness of the crops (Dakora and Keya, 1997; Ayalew and Yoseph, 2020)_._ A possible challenge to the development of a more efficient cowpea rhizobia inoculant with an efficacious BNF potential is the low symbiotic specificity of the crop, leading to the association with a range of indigenous tropical rhizobia with a poor nitrogen-fixation ability (Sena et al., 2020). However, recent developments of rhizobia strains that are capable of associating and fixing biological N_2_ with a large range of hosts, including cowpea, common bean (*Phaseolus vulgaris* L.), and pigeon pea (*Cajanus cajan* (L.), Millsp.), offer a promising avenue for the improvement of the cowpea BNF research (da Silva Júnior et al., 2018; Shamseldin and Velázquez, 2020; Jorrin et al., 2021).

The N_2_ fixation process is highly dependent on P availability and requires the hydrolysis of 16–24 moles of ATP for each mole of N_2_ (Hoffman et al., 2014; Lindström and Mousavi, 2020). A higher inoculation response is predictable and profitable under moderate P rate supplementation to grain legumes (Ronner et al., 2016; Ulzen et al., 2018). In the context of small-scale farming in Africa, many soils are highly deficient in the available P, which results in a low yield of crops (Manyong et al., 2001; Jemo et al., 2015). Other various edaphic factors impair rhizobia development in the soil, in particular soil N availability (Nishida and Suzaki, 2018), soil pH (Yates et al., 2021), drought (Staudinger et al., 2016), salinity (Etesami and Adl, 2020), clay or organic matter content (Thilakarathna and Raizada, 2017), elevated temperature (Basu and Kumar, 2020), on-farm management practices, natural non-effective rhizobacteria in native soils (Iturralde et al., 2019), and “competitor microbes” or parasitic organisms (Costa et al., 2021).

In general, liquid and peat are the two widely used commercial formulations of rhizobia inoculants in the markets (Boonkerd et al., 1978; López-García et al., 2009). The success of inoculated microbes also depends on the formulation and the inoculation methods of viable, cost-effective inoculants with a prolonged shelf life (Xavier et al., 2004; Schoebitz and Roldán, 2013; Strobel et al., 2018). The concentrations of viable bacterial cells at the formulation stage and the minimum rate of cells on coated seeds during sowing time are important to the success of inoculation response (Catroux, 1991; Lupyawi et al., 2000). In Brazilian conditions, where seed inoculation is largely adopted to improve soybean BNF and yield, Hungria et al. (2017) observed that a maximum rate of 1.2 × 10^6^ bacterial cells applied onto the seed is required to achieve the maximum BNF benefit. Furthermore, the minimum bacterial cells for inoculum validation as a commercial standard is ~10^9^ cells g^−1^. However, studies on the influence of inoculum rate on a coated seed and the yield responses to inoculation of grain legumes, such as cowpea and groundnut, have been minimally investigated in Ghana (Naabe et al., 2021). To ensure that grain legumes show a sustainable increase in yield, the present comprehensive review was conducted to critically assess the progress of rhizobia inoculation studies and their management to close yield gaps and increase the profits of the smallholder farmers of Ghana. Specifically, we aimed to:

1. Assess yield variation across the various agroecological zones of cultivation in Ghana.
2. Assess the inoculation responses of grain legumes to rhizobia from experimental studies conducted in Ghana soils.
3. Characterize the extent to which each predictor/explanatory variable contributes to yield variation.
4. Investigate the effects of both combined application of Rhz inoculant and P fertilizer application on yield.
5. Evaluate the effect of bacterial cell concentration and seed coating density to guarantee positive inoculation responses under soil conditions of Ghana.

## Methods

### Literature Search and Database

We conducted a systematic review to assess the effect of rhizobia inoculation in terms of grain yield of three distinct grain legumes (Cowpea, soybean, and groundnut) from field studies in Ghana. Peer-reviewed journal articles were generated on the diverse rhizobia strains tested for each legume, as well as the P application and rhizobia inoculation combination. The Web of Science (webofknowledge.com, United States), Google Scholar (https://scholar.google.com, United States), and Scopus database (https://www.scopus.com/home.uri, Netherland) platforms were used to curate these peer-reviewed journal articles published before December 2021. Four search combinations of peer-reviewed articles were compiled into a single database. An initial search was conducted using the following keywords “Rhizobia inoculation” OR “Biological Nitrogen fixation” OR “Ghana” OR “Grain legumes” OR “Soybean” OR “Cowpea” OR “Groundnut.” In the second search round, our modified keyword terms were as follows: “rhizobia strains” OR “Yield” OR “Biological Nitrogen fixation” OR “Grain legume” OR “Soybean” OR “Cowpea” OR “Groundnut.” Next, to generate articles on the P application, rhizobia inoculations, and combined applications of rhizobia inoculants and P fertilizer for the different grain legumes, our keywords were modified as follows: “Phosphorus application” OR “Rhizobia inoculation” OR “Grain legume” OR “Soybean” OR “Cowpea” OR “Groundnut” OR “Ghana.” To include the rhizobial inoculant formulation methods, the search term was “Rhizobia formulation” OR “Ghana.” A further step toward database construction was to remove redundant articles from the database. A total of 33 publications were retained to compare the effects of Rhz inoculation, P application, both Rhz and P applications, and formulation methods including the bacterial cell concentrations and the seed inoculum rate in the final database. Data were further extracted using the following criteria: (1) studies must include a control treatment without Rhz or P application; (2) if a paper reported multiple independent trials, for example, more than two experiments at a separate location, year, inoculant, P application, and crops, each was recorded as a separate observation in the final dataset. The details regarding the tested grain legumes, origin, rhizobia strains used, inoculation, formulation methods, and reported observations are listed in Supplementary Table S1.

Grain yield was compiled from individual trial reports. The data provided in the tables were manually extracted from each article. For the articles in which data were presented as graphs, the data were extracted with the help of the WebPlot Digitizer (Rohatgi, 2015). Data were standardized to international standard units in kg ha^−1^ to ensure homogeneity. Normalized observations from measured variables were calculated as the percentage increase or decrease over the corresponding uninoculated, non-P application control (Zhang et al., 2021) following Equation 1.


(1) $$\begin{array}{l} \text{Response to inoculation }\\ \;=[(\text{Inoculated value}-\text{Uninoculated value})\;\\ \;/\text{Uninoculated value}]\times 100 \end{array}$$

### Statistical Analysis

The Nelder–Mead method was used to estimate the sample effect sizes for the tested parameters (Nelder and Mead, 1965). The minimum, mean, maximum, and sample size (*n*) values were calculated using the JMP of Statistical Analysis Software (SAS, version 14.3.0) (JMP, 2019) in the different tables. Average effect size and corrected bias at 95% confidence intervals (CI) for each grain of legumes were calculated using a mixed-effect model using Origin-Pro (2020).

Permutational multivariate analysis of variance (PERMANOVA) based on the Bray-Curtis distance matrix was used to extract the effects of the different factorial predictors on the grain yield change using the Vegan package in R software (R version 4.0.2, 2020). A non-metric multidimensional scaling (NMDS) was used to visualize the various predictors, principally agroecological zones (AEZ), genotypes (Gen), rhizobia strains (Rhz strains), P application (P appl), rhizobia × P application (Rhz and P appl.), and formulation methods (Formula) using the metaMDS function in the Vegan package. A redundancy analysis (RDA) was further performed to identify the main factors that predict the yield change under Ghana soil conditions. A constrained RDA with 500 permutations was conducted to separate the effect of each predictor on grain yield. Only predictors with significant effect at *p* < 0.05 are reported in graphs. A “random forest” regression model in the R package (Version 4.0.2, 2020) was used to compute variables of relative importance for yield variation for each grain legume crop.

## Results

### Yield Variation Across Agroecological Zones (AEZ) of Ghana

**Figure 1:**
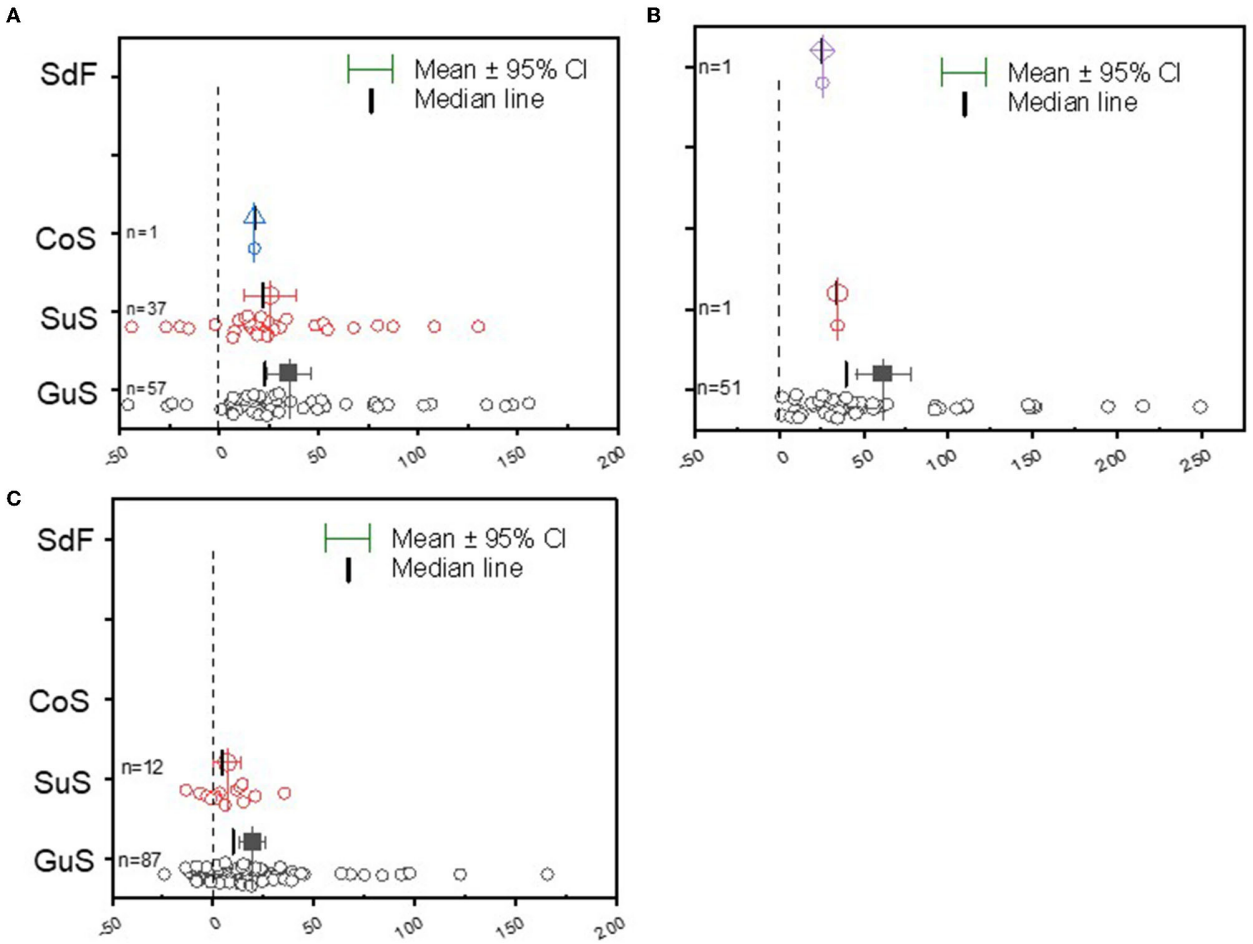
Boxplot and scatter data (open circle symbol) of the relative increase in grain yield of **(A)** soybean, **(B)** cowpea, and **(C)** groundnut to rhizobia inoculation compared to the uninoculated control plants in the Guinea savanna (GuS), Sudan savanna (Sus), Coastal savanna (Cos), and semi-deciduous forest (SdF) agroecological zones of Ghana. The mean values (different symbols) with a 95% bootstrap confidence interval, the median line, and the sample size (*n*) are shown.

The meta-analysis results aggregated from the different AEZ of grain legumes are shown in Figure 1. For the different legume crops, the Guinea savanna represents the predominant domain in which most field studies on grain legumes are conducted in Ghana (Figures 1A–C). The Sudan savanna constitutes the second domain of soybean and groundnut cultivation (Figures 1A,C). The average yield response was higher in the Guinea savanna than in the Sudan savanna soil conditions for soybean, cowpea, and groundnut, and the data distribution ranges were positively (right) skewed under the Guinea savanna soils (Figures 1A–C). The data regarding yield distribution range was also right-skewed, but less dispersed in the Sudan savanna field conditions, implying that the average yield was higher than the median value (Figures 1A,C).

### Response of Grain Legumes to Rhizobial Inoculants

**Figure 2:**
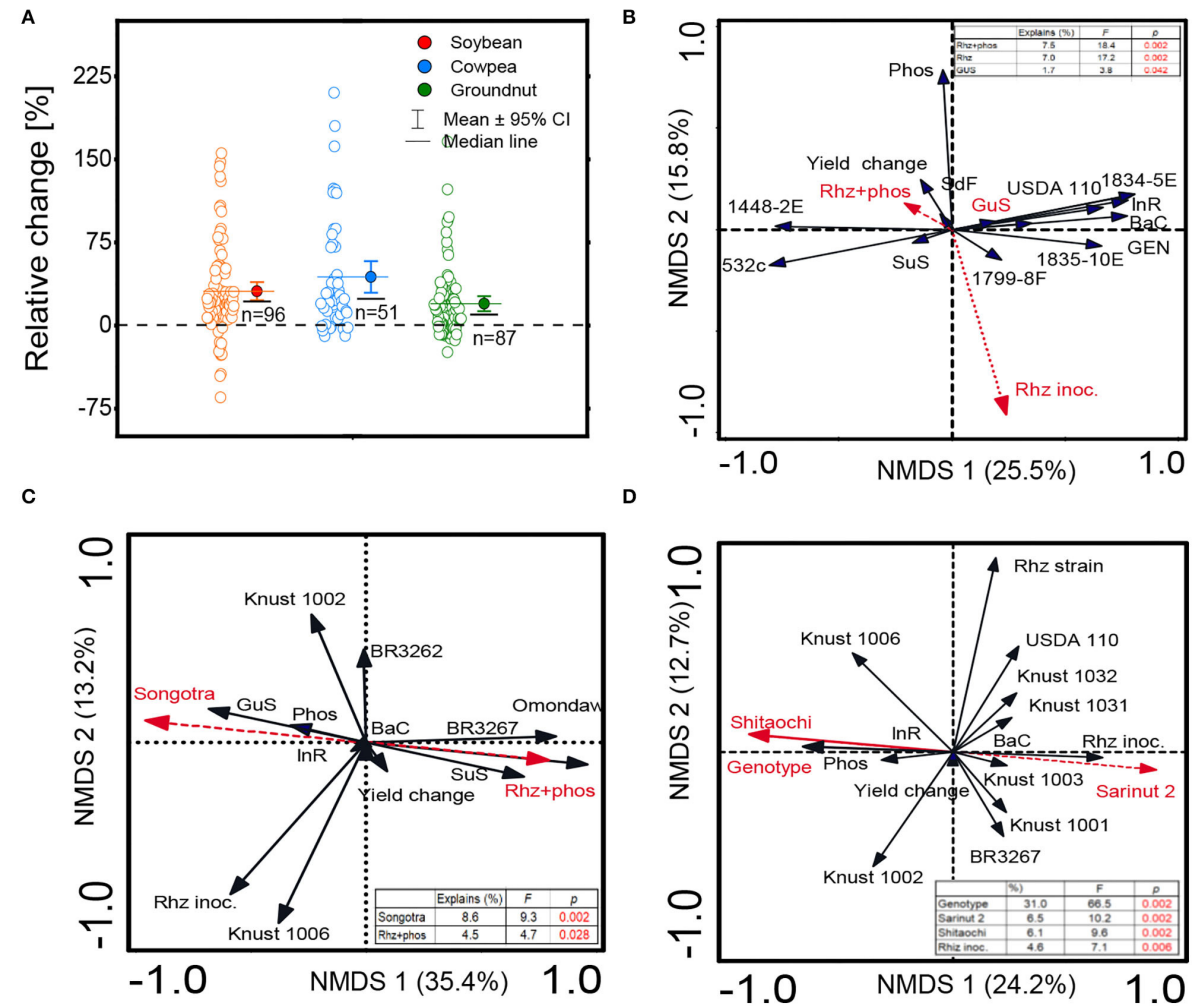
Boxplot and scatter data (open circle symbol) of **(A)** the relative increase in grain yield of soybean, cowpea, and groundnut inoculated with diverse rhizobium strains compared to the uninoculated control plants under Ghanian soils and non-metric multidimensional scaling (NMDS) depicting the effect on **(B)** soybean, **(C)** cowpea, and **(D)** groundnut grain yield of sets of variables relative to the plant genotype, rhizobium strains, and phosphate fertilizer application. The mean values (different symbols) with a 95% bootstrap confidence interval, the median line, and the sample size are shown. Guinea savanna (GuS), Sudan savanna (SuS), Coastal savanna (CoS), semi-deciduous forest (SdF), genotype (Gen), TGx 1834-5E (1834-5E), TGx 1799-8F (1799-8F), TGx 1448-2E (1448-2E), TGx 1835-10E (1835-10E), bacterial cell concentration (BaC), inoculum rate (InR), phosphorus application (Phos), rhizobium inoculation (Rhz inoc.), and rhizobium strains (Rhz strain).

The detailed information regarding the genotypes of soybean, cowpea, and groundnut, the origin of rhizobia species, commercial names, formulation types, and sample size are reported in Supplementary Table S1. Irrespective of the genotypes or the rhizobial species/strains tested, the average grain yield responses to rhizobial inoculation were positive in the three grain legumes (Figure 2A). The boxplots of the distribution range of data exhibited the highest average inoculation response (61.7%) for cowpea plants and the lowest (19.8%) for groundnut. The data distribution range was right-skewed for all the three crops, that is, soybean, cowpea, and groundnut (Figure 2A). The yield responses to inoculation were 1.6-, 1.5-, and 1.2-fold higher in rhizobia-inoculated plants than in the non-inoculated soybean, cowpea, and groundnut plants, respectively (Table 1).

**Table 1:** Grain yield (kg ha^−1^) of uninoculated (-Rhz), no P application (no-P), -Rhz-no-P and the rhizobia (-Rhz) inoculated, P (+P) application, and +Rhz+P combination in soybean, cowpea, and groundnut plants under field condition in Ghana.

		**-Rhz**	**+Rhz**	**No-P**	**+P**	**-Rhz and No-P**	**+Rhz+P**
		**[kg ha** ^**−1**^ **]**					
Soybean	Maximum	2,700	3,120	2,770	3,350	2,700	3,485
	Mean	831	1,048	880	1,052	696	1,244
	Minimum	56	336	55	85	55	137
	Sample size (*n*)	96	96	90	90	73	73
Cowpea	Maximum	1368	2,232	2,150	3,600	800	1,900
	Mean	845	1,249	1,021	1,332	664	1,521
	Minimum	175	410	175	425	520	1,200
	Sample size (*n*)	51	51	96	96	7	7
Groundnut	Maximum	1,770	2,377	3,549	4,706	1,450	1,860
	Mean	1,067	1,232	1,090	1,394	1,212	1,704
	Minimum	305	745	305	580	1,085	1,555
	Sample size (*n*)	87	87	60		12	12

**Figure 3:**
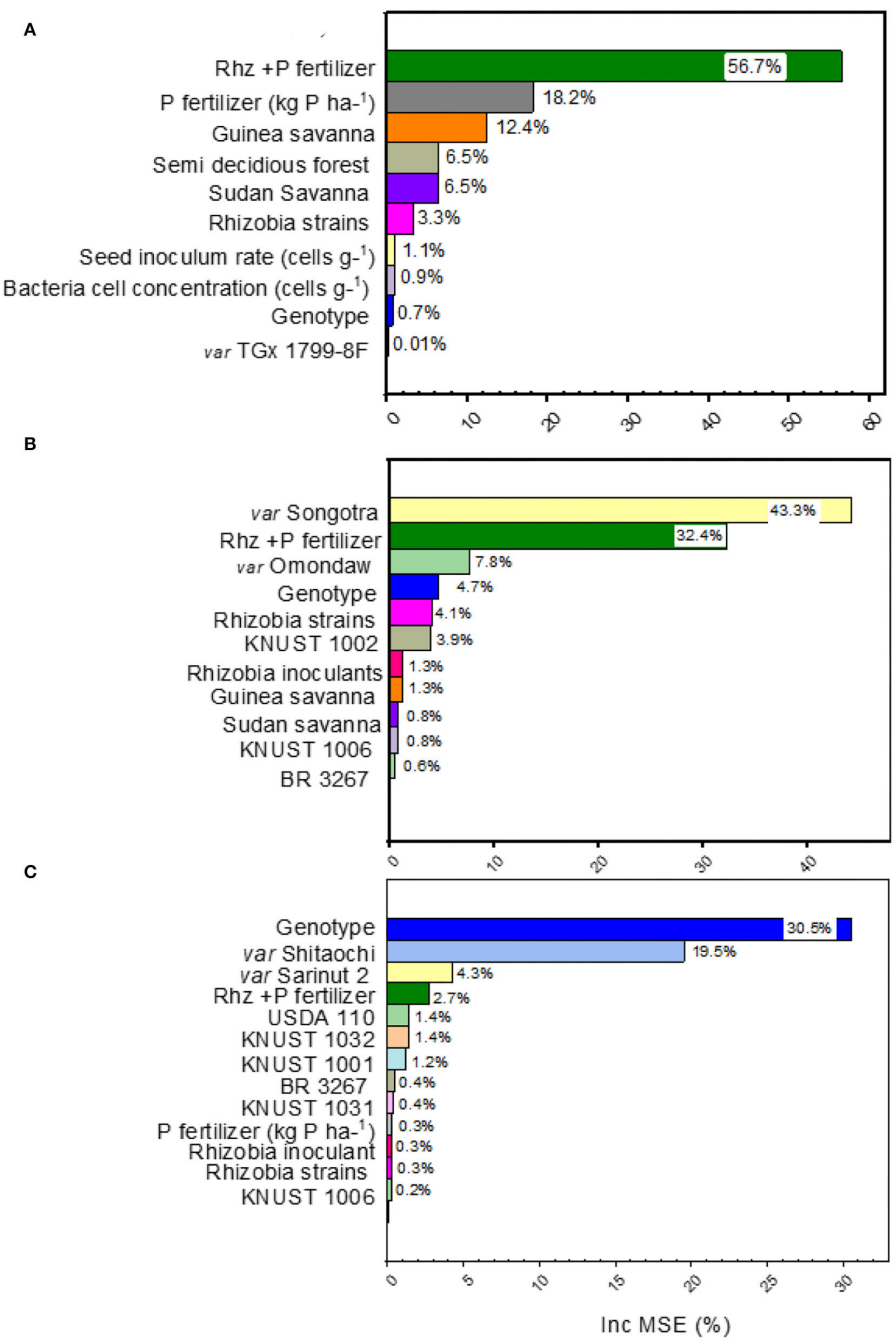
Random forest results of the relative importance of different explanatory variables (rhizobia strains, legume genotype, agroecology, P application, and combined rhizobia inoculant and P application) in explaining variation to “grain yield” change of **(A)** soybean, **(B)** cowpea, and **(C)** groundnuts from field experimental studies in Ghana included in the present meta-analysis.

The range of yield changes and their predictors varied across different species of grain legumes (Figures 1B,C, 3A–C). The NMDS and RF regression model used to quantify the determinants of grain yield variation revealed that the combination of Rhz and P application (7.5%), rhizobia inoculants alone (7%), and Guinea savanna (1.7%) agroecology explained the largest percentage of yield change for soybean according to the Fisher (F) values reported (*p* < 0.05) (Figure 2B). The machine learning and data-mining algorithm disclosed that the Rhz + P fertilizer combination (56.7%), P fertilizer (18.2%), and the Guinea savanna (12.4%) are highly important explanatory variables of yield variation in soybean (Figure 2A). For cowpea, the genotype Songotra and the combination of Rhz and P fertilizer were the important explanatory predictors of yield change, contributing to 8.6% (*p* = 0.002) and 4.7% (*p* = 0.028), respectively (Figure 2C). The RF regression model of the relative importance of variables contributing to cowpea yield change is shown in Figure 3B. The genotype Songotra and the combined application of Rhz + P fertilizer treatments were relatively important variables, with 44.3 and 32.4% contributions, respectively (Figure 3B). Several explanatory variables were responsible for the variation in the groundnut yield, particularly the tested groundnut varieties (31.1%), varieties Sarinut-2 (6.5%) and Shitaochi (6.1%), and rhizobia inoculant (4.6%). The groundnut genotypes (30.1%), varieties Shitaochi (21%) and Sarinut-2 (4.3%), and the combined application of Rhz and P fertilizer (2.7%) made the highest contribution to yield variation in groundnut (Figure 3C).

### Yield Responses to Inoculated Rhizobia Strains

**Figure 4:**
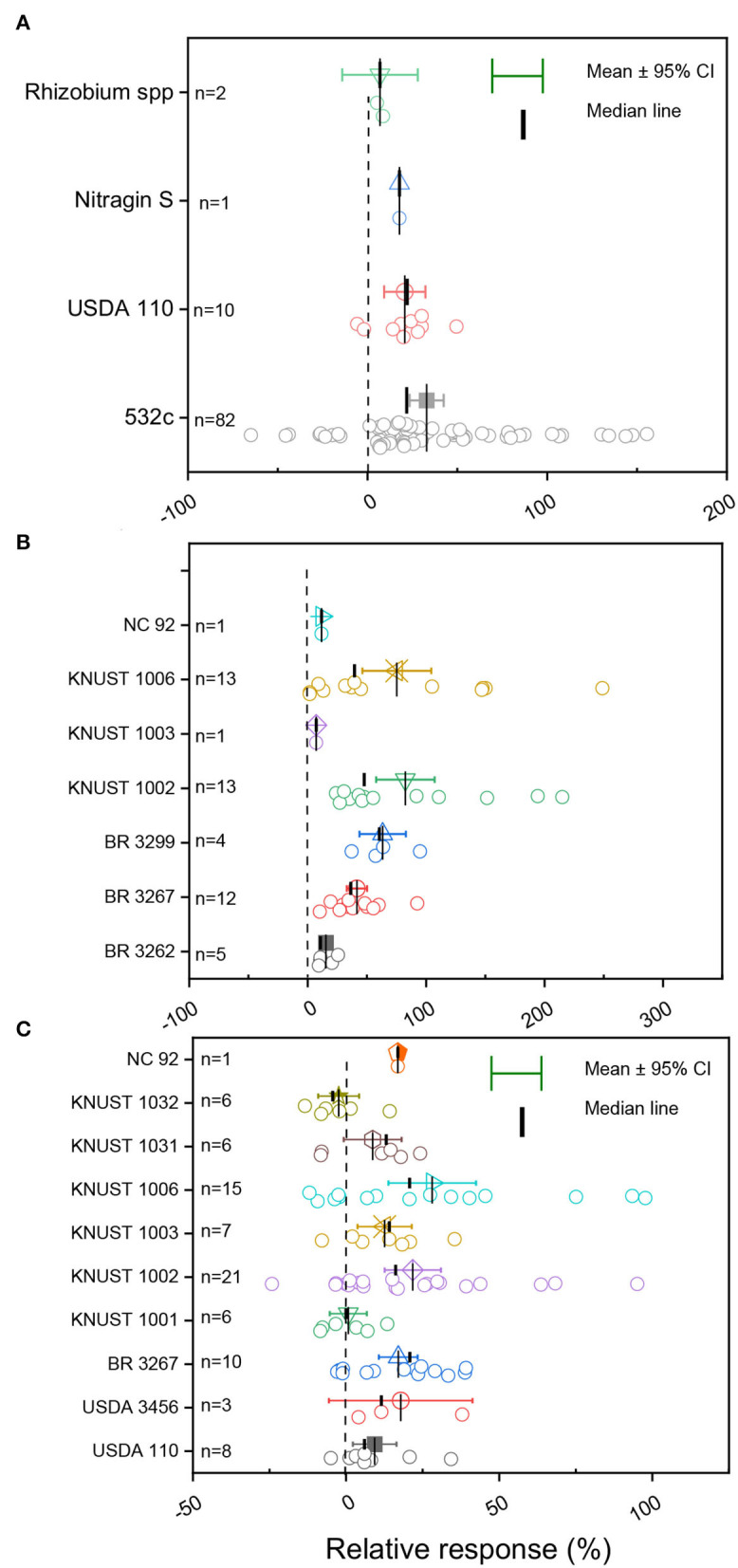
Boxplot and scatter data (open circle symbol) of the relative increase in grain yield following inoculation with diverse rhizobia strains for **(A)** soybean, **(B)** cowpea, and **(C)** groundnut compared to the uninoculated control plants under Ghanian soil. The mean values (different symbols) with a 95% bootstrap confidence interval, the median line, and the sample size are shown.

The details of the diverse rhizobia strains tested on the different legume crops are presented in Supplementary Table S1. For soybean plants, three strains of *B. japonicum* (532c, USDA 110, and Nitragin-S) and one strain of *Rhizobium* spp. isolated from various origins were tested in Ghana (Figure 4; Supplementary Table S2). The average grain yield change depicted a tightly clustered and normal distribution of data for USDA 110, while a dispersed and negative distribution of observations was noticed for cowpea plants inoculated with rhizobia strain 532c (Figure 4A). In our curated data, only one study used the strain Nitragin-S (Figure 4A).

The meta-analysis in cowpea reported three native (KNUST 1002, KNUST 1003, and KNUST 1006) and four imported strains of *B. pachyrhizi* (BR 3262), *B. yuanmingense* (BR 3267), *Microvirga vignae* (BR 3299), and *Bradyrhizobium* sp. (NC 92) out of the seven rhizobia strains tested in the different ecological zones of Ghana (Supplementary Table S2). A positive grain yield change for inoculated cowpea plants compared to the non-inoculated plants was reported for all the tested Rhz strains, except for strain NC 92 (Figure 4B). The yield responses to the inoculated Rhz strains were skewed with a dispersed and negative distribution for strains KNUST 1006 and KNUST 1002 (Figure 4B). Conversely, we reported tightly clustered and normally distributed observations for strains BR 3299, BR 3267, and BR 3262 (Figure 4B). The highest yield change response was reported for strain KNUST 1006 (82.5%), while strain KNUST 1003 (7.2%) induced the lowest yield change response to inoculation (Figure 4B).

In groundnut plants, 10 rhizobia strains were tested, of which six strains were locally isolated (KNUST 1001, KNUST 1002, KNUST 1003, KNUST 1006, KNUST 1031, and KNUST 1032) and four strains were introduced (USDA 3456, USDA 110, BR 3267, and NC92) (Figure 4C; Supplementary Tables S1, S2). Inoculation with the diverse rhizobia strains resulted in a positive yield change, except for strain KNUST 1032 (Figure 4C). The various observations reported for the different rhizobia strains were dispersed, except for strain KNUST 1001, which was normally distributed but showed only a small yield change response. The highest average yield change due to the inoculation was observed for strain KNUST 1006 (28.1%) and the lowest change for KNUST 1032 strain (−2.4%) (Figure 4C).

### Rhizobia Inoculant and P Combination to Improve Grain Legume Yield

**Figure 5:**
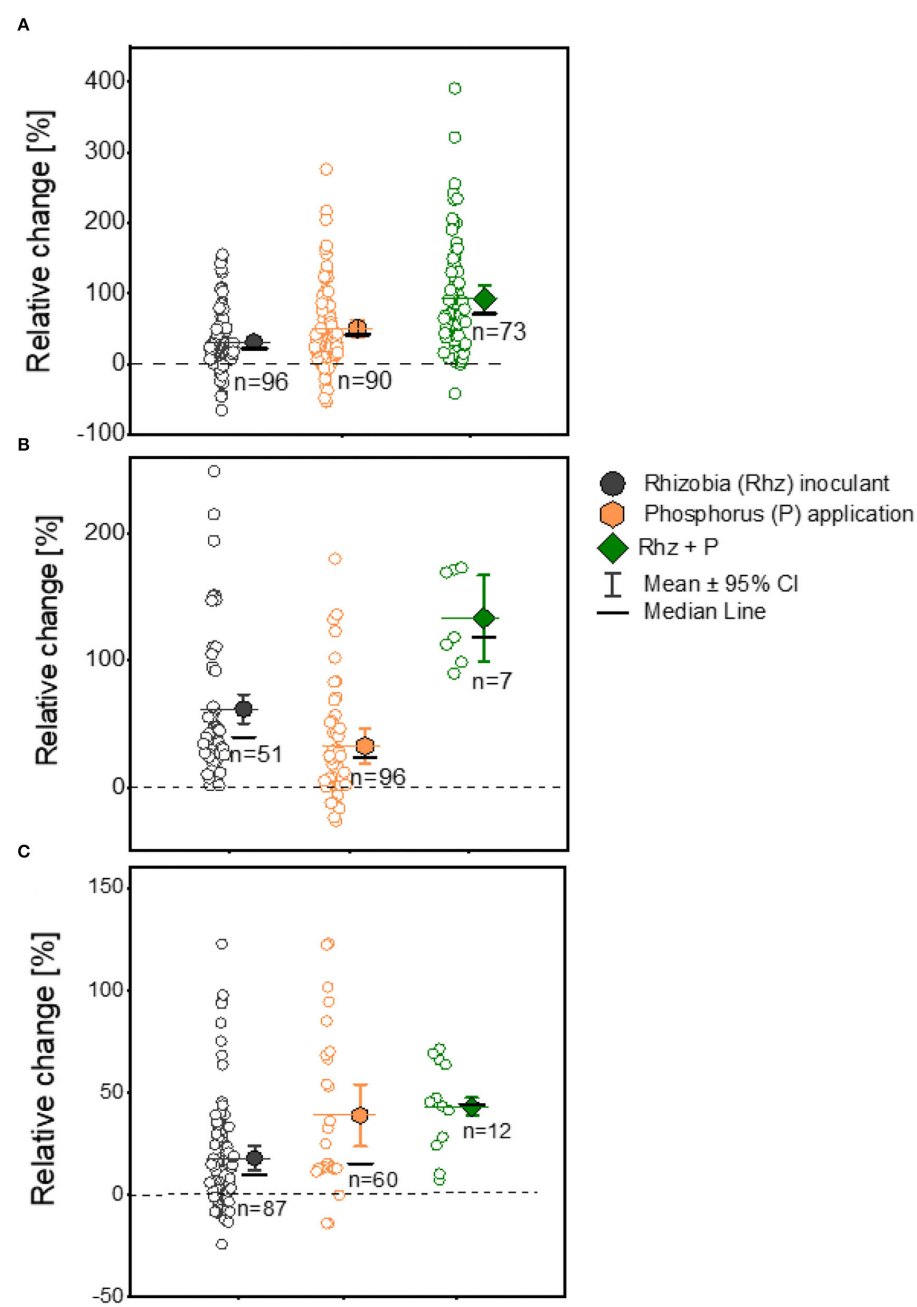
Boxplot and scatter data (open circle symbol) of grain yield responses to rhizobia (+Rhz) inoculation, phosphorus (+P) application, and +Rhz+P over the uninoculated (-Rhz), no-P, and -Rhz-no-P treatments for **(A)** soybean, **(B)** cowpea, and **(C)** groundnut plants under Ghana soil. The mean values (different symbols) with a 95% bootstrap confidence interval, the median line, and the sample size are shown.

We aggregated data from the experimental studies involving three legumes with Rhz inoculation with/without P supplementation or Rhz inoculation but with P supplementation to investigate whether biological and mineral inputs in combination could improve the legume yield in smallholder farms in Ghana. The combined application of Rhz + P fertilizer exhibited the highest (92.6%) yield response compared to Rhz inoculation (31.0%) and P application (50.9%) alone for soybean plants, despite a dispersed distribution of the recorded observations (Figure 5A; Table 1). The Rhz + P application treatment also displayed a higher yield change response (133.3%) than the Rhz (61.7%) and P supplement (32.6%) alone for cowpea plants (Figure 5B; Table 1). We further noticed a tight and normal distribution of observations following the Rhz **+** P application treatment for cowpea plants (Figure 5B). For groundnut plants, the Rhz **+** P application also showed a higher (43.1%) yield response than the Rhz inoculation (19.8%) and P supplementation (38.9%) alone (Figure 5C). The yield increase ratios in the (Rhz **+** P)/Rhz and (Rhz**+** P)/P treatments were 1.21- and 1.24-fold higher, respectively, in the Rhz + P treated soybean plants (Table 1). Similarly, the yield increase ratios were 1.17- and 1.1-fold in favor of the Rhz + P treatment in cowpea plants (Table 1). We reported a yield increase ratio of about 1.4- and 1.22-fold in the Rhz **+** P/ Rhz and Rhz**+** P/P treatments, respectively, in the groundnut plants (Table 1).

### Grain Yield Response to Rhz Inoculant Cell Concentration and Seed Inoculum Rate

**Figure 6:**
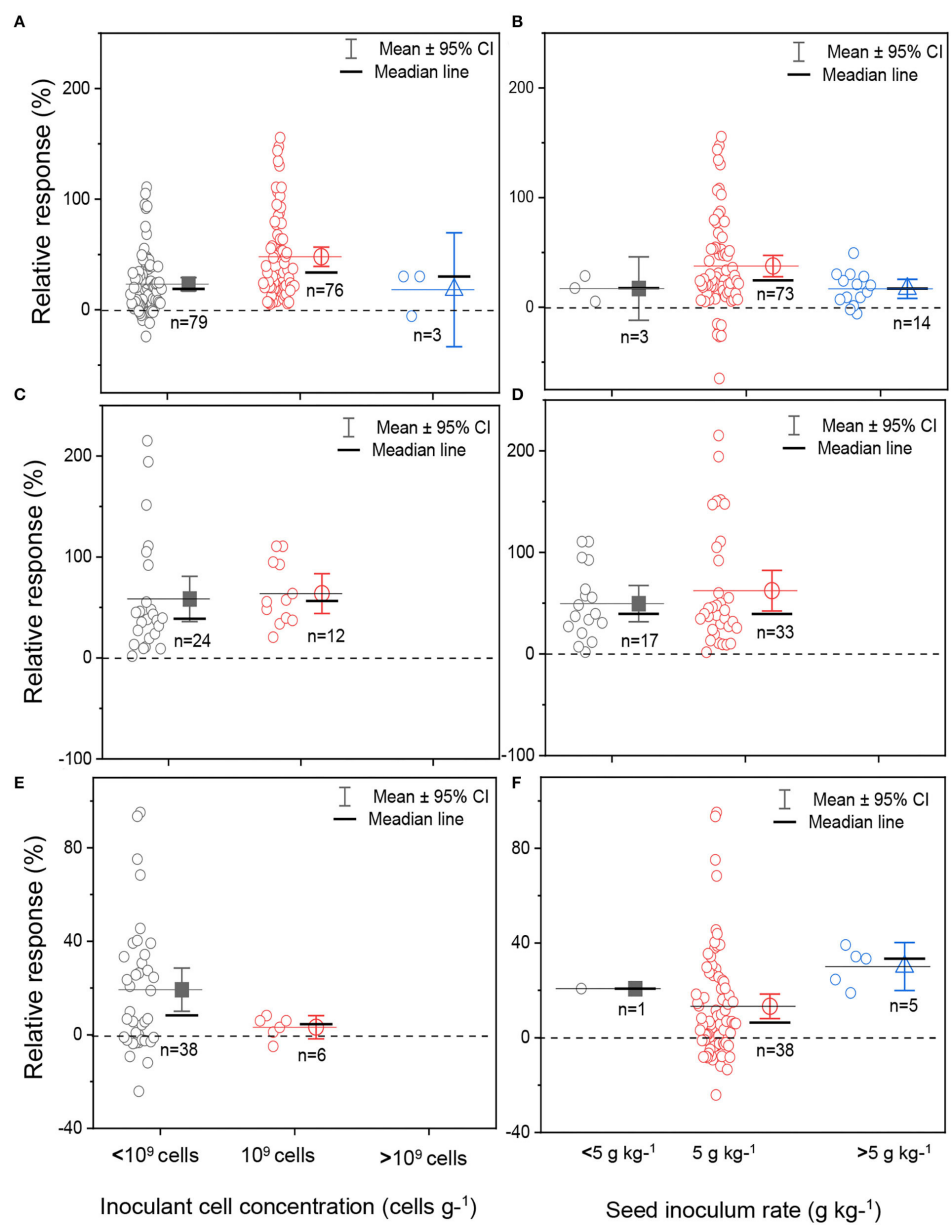
Boxplot and scatter data (open circle symbol) of soybean **(A,B)**, cowpea **(C,D)**, and groundnut **(E,F)** grain yield responses to bacterial cell inoculant concentration **(A,C,E)** and inoculant seed coating density **(B,D,F)** under Ghana soil conditions. The mean values (different symbols) with 95% bootstrap confidence interval (CI), the median line, and the sample size are shown.

The boxplots and scatter data for bacterial cell concentration at the formulation stage and the seed inoculum rate are shown in Figure 6. Three classes of bacterial cell concentrations in formulations (<10^9^ cells, ~10^9^ cells, and >10^9^ cells) and seed inoculum rates (<5, 5, and >5 g kg^−1^) were compared in the meta-analysis. The inoculant formulated with the bacterial cell concentration of 10^9^ cells g^−1^ reported the highest sample size for soybean crop (Figure 6A). The data distribution was skewed toward the right (positive) for bacterial cell concentration below 10^9^ cells g^−1^ and skewed toward the left (negative) for inoculant cell concentration above 10^9^ cells g^−1^ for soybean (Figure 6A). For cowpea, the median lines of the yield responses between the concentration <10^9^ cells and 10^9^ cells were at the same level, implying that there were no statistical differences between each of the two inoculant cell concentrations (Figure 6C). For groundnut, we observed a lower yield response in the inoculant formula with a bacterial cell concentration of 10^9^ cells than that containing <10^9^ cells (Figure 6E).

The seed inoculum rate of coated rhizobia inoculants varied from 3 g kg^−1^ of seed to 10 g kg^−1^ of seed for soybean, cowpea, and groundnut plants (Figures 6B,D,F). Most experiments used an average rate of 5 g kg^−1^ of seed, corresponding to 1.2 × 10^6^ cells seed^−1^, which facilitated a higher yield response for soybean, but not for cowpea and groundnut (Figures 6B,D). Inoculum rate above 5 g kg^−1^ of seed gave the highest average yield response, while the seed inoculum rate of 5 g kg^−1^ resulted in the lowest average yield response for groundnut (Figure 6F).

## Discussion

Raising crop yield to meet food demand and simultaneously increasing the profitability for farmers remain a challenge in the low-input farming systems of Africa (Tian and Yu, 2019). Agricultural technologies focusing on productivity increase, system durability, and improved farmer profitability are needed to enhance the household food security of smallholders (Helfenstein et al., 2020). Grain legume crops, such as soybean, cowpea, and groundnut, are an integral component in many cropping systems due to their capability to biologically fix atmospheric N_2_ and convert it into usable forms of N for the benefit of agricultural systems. The comprehensive research objective of this study was to review grain legume yield variability as a function of legume genotype × inoculant × P fertilizer supplementation using a meta-synthesis of a literature-based data-mining approach based on the studies conducted in Ghana fields. Our analysis generally suggests that a combined and/or integrated technology intervention at a landscape scale, rather than a single approach, is to be undertaken to improve grain legume yield under Ghana farming conditions.

### Legume Yield Variability Across Agroecological Zones of Ghana

A highly dispersed distribution of data was observed in the data collected from the Guinea savanna and Sudan savanna for soybean and cowpea, and a normal distribution range was noticed in the data for groundnut in the Sudan savanna region (Figure 1). There was a tendency for a higher yield response in the Sudan Savanna than in the Guinea savanna regions (Figure 1). Grain legume production areas are predominantly found in the Guinea and Sudan savanna regions (Adjei-Nsiah et al., 2022). The environmental conditions where legumes are cultivated affect the yield due to a combination of factors, such as soil nutrients and climatic conditions (Ulzen et al., 2018; Emmanuel et al., 2021). Poor root nodule development due to the absence of effective rhizobia in the semi-deciduous forest soils impairs the grain yield (Adjei-Nsiah et al., 2022). In the future, selection of the semi-forest deciduous forest zone with two annual growing seasons could lead to an expansion of grain legume cultivation in Ghana with double cropping annually, if appropriate rhizobia strain is also provided to promote nodulation and BNF of the cultivated legume crop.

### Grain Legume Response to Rhizobia Inoculants

These results provide supportive evidence that rhizobia inoculation increases the yield of legume grains with an average increase of 217, 404, and 165 kg ha^−1^ for soybean, cowpea, and groundnut, respectively (Supplementary Table S2). In the absence of the Rhz inoculation, the yield is below 1,000 kg ha^−1^, particularly for soybean and cowpea crops. Grain yield increase in the range of 115–200 kg ha^−1^ was also reported from studies conducted in the United States of America (USA), Argentina, and Africa (Kenya, Nigeria, etc.) for soybean inoculated with *B. japonicum* strain (Leggett et al., 2017; van Heerwaarden et al., 2018). Most of the observations from experimental trials reported positive effects of inoculation. In newly cultivated farm fields, such as in Ghana, inoculation with *B. japonicum* embedded in the Rhz inoculant offers better competitive advantages compared to the native rhizobia and is the principal explanation for the positive yield response in soybean (Rogel et al., 2011). Another plausible explanation is the high selectivity of soybean roots, which nodulate only with *B. japonicum* or *B. diazofficiens* strains. Soybeans discriminate in their selection of bacteria to associate with in symbiotic relationship, preferring *B. japonicum*, by inducing and expressing several dominant genes in their roots to restrict interaction with other Rhz strains (Yang et al., 2010; Tang et al., 2016). The highest relative response to inoculation was observed for cowpea and the lowest for groundnut plants (Figure 2A). It is well-established that cowpea plants have low specificity for rhizobia strains and respond to diverse strains inoculated or available in the local soil (Sena et al., 2020). It is worth mentioning that the cowpea research program of Ghana benefited from extensive research collaboration with Brazilian partners who introduced effective cowpea rhizobia strains, such as BR 3299, BR 3267, and BR 3262, accounting for the observed inoculation responses (Osei et al., 2018, 2020). Groundnut research can benefit from similar collaboration that will help increase the yield through BNF. Efforts are further needed to improve groundnut production to help bridge the current yield gaps (Cernay et al., 2016).

### Determinants of Grain Yield Variability and Potential of Technology Interventions to Close the Legume Yield Gap

We adopted a constrained ordination method to compare the various genotypes (cultivar), rhizobia strains, and inoculant formulations as explanatory (predictor) variables influencing yield change (response variable). A systematic test of the significance of individual predictors to the response variable was implemented for soybean, cowpea, and groundnut (Figures 2B–D). The relative importance of each explanatory variable to grain yield variation was examined (Figure 3). The meta-analysis suggests different contribution patterns for the variables depending on the grain legume crop. For soybean, *B. japonium* was widely used due to its high specificity to nodulate with soybean roots. The combined application with Rhz + P fertilizer and the Rhz inoculant alone were the major drivers of yield change, irrespective of the agroecological zone and soybean genotype (Figure 3B). To maximize the benefit of inoculation and P fertilization in soybean, the integration of highly productive soybean cultivars was also recommended from experimental plot studies in Ghana (Awuni et al., 2020). A non-significant effect of soybean genotype on yield can be attributed to important soybean breeding research programs in Africa, conducted by the International Institute of Tropical Agriculture to develop elite varieties with high productivity across African soils. For cowpea, in addition to combined Rhz + P fertilizer application effects, cultivar Songotra contributed significantly to the yield change (Figure 3C). The result suggests that cowpea breeding in Ghana shall integrate cultivar improvement with Rhz inoculation technology to achieve high grain yield across cultivated agroecological systems. For groundnut, cultivar × rhizobia strain synergistically influenced yield variation (Figure 3C). Among the cultivars, Sarinut-2 and Shitaochi varieties showed the highest explained percentages of relative importance among the groundnut varieties (Figure 3C). The breeding programs in groundnut and Rhz inoculation technologies have not benefited from important research activities regarding the improvement of symbiotic effectiveness on a large scale in contrast to soybean in Africa (Dakora and Keya, 1997; Ayalew and Yoseph, 2020)_._ The aggregated results suggest that combinations between groundnut genotype × improved rhizobia strain × P addition and the local climatic conditions are major factors of yield variation in Ghana. Therefore, research efforts toward addressing these combinations could lead to increased groundnut yield among the smallholders of Ghana.

### Effects of Diverse Rhizobia Strains on Grain Yield

Rhizobia strains from distinctive genetic backgrounds, including three out of the seven tested strains that were imported from Brazil, were inoculated into cowpea plants sown in Ghana (da Silva Júnior et al., 2018; Sena et al., 2020). The distinctive strains were from separate phylogenetic groups (Danso and Owiredu, 1988; Boddey et al., 2016; Osei et al., 2020). On the other hand, locally isolated strains (KNUST 1002 and KNUST 1006) of rhizobia exhibited the highest average yield change for cowpea (Figure 4). Beneficial effects of native rhizobia strains to nodulate and enhance grain yield were also reported in Kenya and Zimbabwe in soybean (Zengeni and Giller, 2007; Waswa et al., 2014). A possible interpretation is that the imported strains did not adapt well to the local soil conditions when compared to the native *Rhizobium* population (Thilakarathna and Raizada, 2017; Ulzen et al., 2018). Another possible reason is the constraint imposed by soil abiotic factors, such as pH, high temperature, clay content, organic matter content, drought, and salinity, which influence the survival of living microbes and result in a poor competition with native rhizobia strains as previously suggested by other authors (Thilakarathna and Raizada, 2017). Apart from a study from Ulzen et al. (2016) that investigated the persistence of *B. yuanmingense* (BR 3267) across a few soil regions of Ghana, there are limited studies on the persistence of introduced vs. local inoculated rhizobia strains and their effects on grain yield. Additional studies on the persistence of the most frequently used rhizobia strain and the need to re-inoculate once introduced are needed. For soybean, all the tested rhizobia species were from the *B. japonicum* and *B. diazoefficiens* groups, irrespective of the soil and edaphic conditions and the origin of strains. The results imply that the success of inoculation is highly determined by the identity of the strain in an inoculant, rather than the origin of the strain used (Fields et al., 2021).

### Combined Application of Rhz Inoculants and P Fertilizer on Yield

The yield response in the Rhz + P treatment was always higher than the Rhz inoculant or P application alone for soybean and cowpea crops (Figure 5, Table 1). The results imply that the application of moderate amounts of P fertilizer (20−30 kg P ha^−1^) and rhizobia inoculants would be profitable for farmers across different soil ecologies of Ghana. The benefits of the combined application of Rhz + P fertilizer were also recorded in common bean, chickpea (*Cicer arietinum* L.), soybean, cowpea, and groundnut in the US, Nigeria, Ethiopia, and other SSA countries (Osman et al., 2002; Mmbaga et al., 2014; Ronner et al., 2016; Wolde-meskel et al., 2018). Phosphorus atoms are central to the N_2_ fixation process, and P deficiency constitutes a severe constraint for adequate N_2_ fixation in legumes (Diaz et al., 2017), particularly in low-P soils of West African regions (Manyong et al., 2001; Jemo et al., 2015; Franke et al., 2018). Generally, the supplementation of P to legume crops enhances the growth and yield, and the N_2_ fixation process is dependent on P, even under unfavorable soil conditions such as drought (Jemo et al., 2017). The yield response to Rhz inoculation alone was comparable to the combined application of Rhz + P fertilizers for cowpea (Figure 5B). Some of the imported rhizobia strains from Brazil probably enhanced the cowpea yield exceptionally due to the previous research efforts to improve the genetic potential of cowpea to perform effective BNF.

### Formulation as a Crucial Step to Field Inoculation Success

Improvements in formulations are key requirements for the development of high-quality inoculants in the marketplace. Inoculant quality is dependent on the formulated bacterial cell concentration, shelf life, inoculum rate per seed, and the cell coating approach to ensure better cell positioning around the seeds, which can further enhance the nodulation process. The results from the meta-analysis showed that most formulated inoculants for soybean, cowpea, and groundnut were below the commercial standard requirements with <10^9^ cells g^−1^ (Figure 6). In Brazil, a minimum bacterial cell concentration of 10^9^ cells is required for the commercialization of rhizobia inoculant to achieve a positive inoculation response for soybean (Bomfim et al., 2021). The highest yield in soybean was observed with the formulated bacterial cell concentration of 10^9^ cells g^−1^ in the inoculant and the inoculum rate of 1.0 × 10^6^ cells seed^−1^ of soybean (Figure 6). The results are in accordance with other inoculation studies conducted in old or newly cultivated soybean fields in Brazil by Hungria et al. (2017). The authors reported that a minimum rate of 1.2 × 10^6^ colony forming units (CFU) seed^−1^ of *B. japonicum* was required to benefit BNF in soybean and obtain a higher inoculation response. For cowpea, the association between the bacterial cell concentration of 10^9^ cells g^−1^ in the case of tested rhizobia inoculants in Ghana and yield, and between the inoculum rate seed^−1^ and yield response is poorly understood. Cowpea often develops a low specificity to rhizobia strains and responds to diverse inoculated or native strains available in the local soil, thus leading to poor BNF benefits (Sena et al., 2020). For groundnut, rhizobia inoculants formulated with a CFU <10^9^ cells g^−1^ showed the highest yield response, while seed inoculum rate above 5 g kg^−1^ also displayed a higher yield response (Figure 6). Previous Rhz inoculation studies on groundnut also observed that rhizobia cell concentrations at 10^5^-10^6^ cells g^−1^ can induce nodulation and fix N_2_ from the atmosphere in certain conditions (Catroux, 1991; Lupyawi et al., 2000). The minimum inoculum rate required per seed has only received little research attention in Ghana. The results call for further investigations to determine the rhizobia inoculum standard, the required inoculation rate of bacterial cells that guarantee a higher yield of groundnut, and maximum profits for small farmers.

## Conclusion

By aggregating different field experimental studies conducted in Ghana on Rhz inoculation, P application, combined application of Rhz + P fertilizers, and incorporating the diverse legume genotypes, Rhz strains, as well as the geographic climatic references, the present study demonstrated that cowpea showed the highest average yield change and groundnut showed the lowest. Combined application of Rhz inoculation + P fertilizers contributed to a relatively high proportion (73.3%) of yield change of soybean across the various agroecological zones, while the incorporation of cowpea genotype together with combined application of Rhz + P fertilizer accounted for the maximum yield variation in that crop. For groundnut, various predictors influenced the yield change, particularly the combined Rhz + P fertilizer, inoculation of Rhz strain (KNUST 1031) alone, genotype (Sarinut−2 and Shitaochi), and soil agroecology. The significant effects of soil agroecology, legume genotype, P management, and Rhz inoculation on yield change suggest that an integrated technological set of interventions are necessary for durable yield enhancement in legumes. Integrated inputs, including high-quality rhizobia inoculants and improved seed varieties guided by good management practices with respect to P supplementation, will lead to increased legume yield, reduced yield gaps, generation of profit, and maintenance of environmental sustainability indicators. For cowpea and groundnut, prospective studies to investigate the minimum requirement of bacteria cells g^−1^ in the formulated inoculants and minimum inoculum rate seed^−1^ are required for optimum inoculation response and benefits.

Possible gaps in the research methodology to consider during the interpretation of results could be a meta-synthesis approach to compare data or observations from separate independent studies, set up, and performed by scientists with different expertise. However, a PERMANOVA analysis coupled with a random forest regression model and the Nelder–Mead method estimated the weighted average effects and mitigated the heterogeneity, and increased the interpretability of the aggregated data. The real advantage of the meta-analysis approach is that it allows to combine and synthesize data from multiple independent studies and integrate the results to improve decision making in a largescale intervention trial.

## Data Availability Statement

The original contributions presented in the study are included in the article/Supplementary Materials, further inquiries can be directed to the corresponding author/s.

## Author Contributions

ABB and MJ: writing original draft. ABB, MK, NB, JJ, MA, YO, MH, and MJ: methodology. ABB, MK, NB, JJ, MA, YO, MH, SD, AB, and MJ: manuscript review and inputs. All authors contributed to the article and approved the submitted version.

## Funding

This study received funding from OCP Africa through a grant no 001 to funded to UM6P. The funder was not involved in the study design, collection, analysis, interpretation of data, the writing of this article, or the decision to submit it for publication.

## Conflict of Interest

AB and SD were employed by the company OCP Africa, but OCP Africa had no role in the study design, data collection and analysis, decision to publish, or preparation of the manuscript. The remaining authors declare that the research was conducted in the absence of any commercial or financial relationships that could be construed as a potential conflict of interest.

## Publisher's Note

All claims expressed in this article are solely those of the authors and do not necessarily represent those of their affiliated organizations, or those of the publisher, the editors and the reviewers. Any product that may be evaluated in this article, or claim that may be made by its manufacturer, is not guaranteed or endorsed by the publisher.
