# Supplementary material for: Grain Legume Yield Responses to Rhizobia Inoculants and Phosphorus Supplementation Under Ghana Soils: A Meta-Synthesis
Source: Front Plant Sci. 2022 Jun 23;13:877433. doi: 10.3389/fpls.2022.877433 (PMC9261782; doi:10.3389/fpls.2022.877433)
Supplement: Supplementary file 1 [file Data_Sheet_1.docx]

Grain legume yield responses to rhizobia inoculants and phosphorus supplementation under Ghana soils: A meta-synthesis

Alfred Balenor Buernor ^1^ • Muhammad Rabiu Kabiru^1,2^ • Noura Bechtaoui^1^• Jibrin Mohammed Jibrin^2^ • Michael Asante^3^ • Anis Bouraqqadi^4^ • Sara Dahhani^4^ • Yedir Ouhdouch ^1,5^ • Mohamed Hafidi^1,5^ • Martin Jemo^1,^ *

Supplementary information

Supplementary Table 1. Tested grain legumes, Rhizobia (Rhz) strains, commercial name, inoculant origin, reported observations, and nature of experiments under each independent factors (Rhizobia inoculation (Rhz), P application (P appl), combined applications of Rhz+P, formulation and application methods, and cited references under Ghana soil conditions

| Legume | Genotype and registration code in Ghana | Rhizobium (Rhz) strain | Rhizobium specie | Commercial name | Strain’s origin | Sample size reported | | | References |
| --- | --- | --- | --- | --- | --- | --- | --- | --- | --- |
|  |  |  |  |  |  | Rhz | P appl | Rhz+P |  |
| Cowpea | Songotra (GH/Vu/006/15) | BR 3267 | *B. yuamingense* | SARIFIX | Brazil | 3 | 3 | 3 | Ulzen et al. 2019. |
|  | Padi-Tuya (GH/Vu/005/15) | BR 3299 | *Microvirga vignae* |  | Brazil | 16 | 0 | 0 | Boddey et al. 2016 |
|  |  | BR 3262 | *B. pachyrhizi* |  |  |  |  |  |  |
|  | Songotra (GH/Vu/006/15) | KNUST 1002 KNUST 1006 | *Bradyrhizobium sp.* | - | Ghana | 24 | 0 | 0 | Osei et al. 2020 |
|  | Wang-Kae (GH/Vu/007/16) | KNUST 1002 | *Bradyrhizobium sp.* | - | Ghana | 5 | 0 | 0 | -  Mintah et al. 2020 |
|  |  | KNUST 1006 |  |  |  |  |  |  |  |
|  |  | KNUST 1006 |  |  |  |  |  |  |  |
|  |  | BR 3267 | *B. yuamingense* |  | Brazil |  |  |  |  |
|  |  | NC 92 | *Bradyrhizobium sp.* |  |  |  |  |  |  |
|  | Asomdwee GH/Vu/010/15)  Songotra (GH/Vu/006/15) | BR 3262 | *B. pachyrhizi* | - | Brazil | 1 | 0 | 0 | Miheretu & sarkodie-addo 2017 |
|  | Omondaw (not registered) | BR 3267 | *B. yuamingense* | SARIFIX | Brazil | 4 | 0 | 8 | Emmanuel et al. 2021 |
|  | - |  | *B. japonicum* |  | Brazil | 0 | 3 | 0 | Adjei-Nsiah et al. 2018 |
|  | Asomdwee (GH/Vu/010/15)  GH-2200  GH-2309  F2T2K66  GH-6060  GH-5344  Hans adua (GH/Vu/001/16)  Nketewade (GH/Vu/003/16)  F2T2A36 | - | - | - | - |  | 20 | 0 | Adusei et al. 2020 |
|  | Asontem (GH/Vu/010/15) | - | - | - | - | 0 | 2 | 0 | Ofosu-budu et al. 2007 |
|  | IT × P 148 | - | - | - | - | 0 | 3 | 0 | Karikari et al. 2015 |
|  | IT89KD-347-57 |  |  |  |  | 0 | 3 | 0 |  |
|  | Asetenapa |  |  |  |  | 0 | 3 | 0 |  |
|  | Asomdwee (GH/Vu/010/15) |  |  |  |  | 0 | 3 | 0 |  |
|  | Valenga (GH/Vu/001/15) |  |  |  |  | 0 | 3 | 0 |  |
|  | Bengpla (GH/Vu/002/15 |  |  |  |  | 0 | 3 | 0 |  |
|  | Padi-Tuya (GH/Vu/005/15) | - | - | - | - | 0 | 3 | 0 | Atakora et al. 2014 |
|  | Asontem (GH/Vu/010/15) | - | - | - | - | 0 | 3 | 0 | Daramy et al. 2017 |
| Soybean | TGx 1448-2E (GH/Gm/005/15) | - | Rhizobium sp. |  |  | 4 | 4 | 4 | Avornyo et al. 2020 |
|  | TGx 1448-2E (GH/Gm/005/15) | 532 c | *B. japonicum* | Legumefix | UK | 181 | 181 | 181 | Ulzen et al. 2018 |
|  | TGx 1448-2E (GH/Gm/005/15 | USDA 110 | *B. diazoefficiens* | Biofix | UK | 5 | 0 | 0 | Ulzen et al. 2016 |
|  |  | 532 c | *B. japonicum* | Legumefix | UK |  |  |  |  |
|  | TGx 1448-2E (GH/Gm/005/15) | 532 c | *B. japonicum* | Legumefix | UK | 2 | 0 | 0 | Masso et al. 2016 |
|  | CES 486 | Nitragin S | *B. japonicum* | - |  | 4 | 4 | 0 | Dadson & Acquaah, 1984 |
|  | TGx 813-6D (GH/Gm/003/15)  TGx 1448-2E (GH/Gm/005/15)  TGx 1799-8F (GH/Gm/011/15)  TGx 1835-10E (GH/Gm/001/16) | 532 c | *B. japonicum* | Legumefix | UK | 5 | 0 | 0 | van Heerwaarden et al. 2018 |
|  | - | USDA 110 | *B. diazoefficiens* |  | USA | 59 | 59 | 59 | Adjei-Nsiah et al. 2018 |
|  | TGx 1834-5E (GH/Gm/009/15)  TGx 1448-2E (GH/Gm/005/15) TGx 1445-2E (GH/Gm/006/15)  TGx 1445-3E (GH/Gm/010/15)  TGx 1799-8F (GH/Gm/011/15) | USDA 110 | *B. diazoefficiens* |  | USA | 2 | 2 | 2 | Awuni et al. 2020 |
|  | TGx 1448-2E (GH/Gm/005/15)  TGx 1445-2E (GH/Gm/006/15)  TGx 813-6D (GH/Gm/003/15) |  | *B. japonicum* |  |  | 2 | 2 | 2 | Lambon et al. 2018 |
|  | TGx 1448-2E (GH/Gm/005/15) | 532c | *B. japonicum* | Legumefix | UK | 3 | 3 | 3 | Ahiabor et al. 2014 |
|  | TGx 1448-2E (GH/Gm/005/15)  Anidaso (GH/Gm/003/15)  TGx 1445-2E (GH/Gm/006/15) | 532c | *B. japonicum* |  | UK | 2 | 2 | 0 | Aziz et al. 2016 |
|  | TGx 1448-2E (GH/Gm/005/15) |  | *B. japonicum* | SARIFIX |  | 1 | 1 | 1 | Anani et al. 2021 |
|  | TGx 1448-2E (GH/Gm/005/15) | 532c | *B. japonicum* | Legumefix | UK | 3 | 3 | 3 | Ezekiel-Adewoyin et al. 2017 |
| Groundnut | Obolo (GH/Ah/013/15)  Oboshie (GH/Ah/015/15)  Sarinut 2 (GH/Ah/002/19) | USDA 3456 |  |  | USA | 6 | 12 | 12 | Asante et al. 2020 |
|  |  | BR 3267 | *B. yuamingense* |  | Brazil |  |  |  |  |
|  | Shitaochi (GH/Ah/001/15) | KNUST 1002, KNUST 1006 |  |  | Ghana | 28 | 0 | 0 | Osei et al. 2020 |
|  | Nkatiesari (GH/Ah/005/15) | KNUST 1002, KNUST 1006, KNUST 1003 | *Bradyrhizobium sp.* |  | Ghana | 5 | 0 | 0 | Mintah et al. 2020 |
|  |  | NC 92 | *Bradyrhizobium sp.* |  |  |  |  |  |  |
|  | Nkatiesari (GH/Ah/005/15) | USDA 110 | *B. diazoefficiens* | Nodule max | USA | 2 | 2 | 0 | Naabe et al. 2021a |
|  | Shitaochi (GH/Ah/001/15)  Nkatiesari GH/Ah/005/15) | BR 3267 | *B. japonicum* | SARIFIX | Brazil | 6 | 6 | 6 | Naabe et al. 2021b |
|  | Shitaochi (GH/Ah/001/15)  Sarinut 2 (GH/Ah/002/19) | KNUST 1001, KNUST 1002, KNUST 1003, KNUST 1031, KNUST 1032 | *Bradyrhizobium sp.* | - | Ghana | 42 | 0 | 00 | Wilson et al. 2021 |
|  |  | BR 3267 | *B. yuamingense* | SARIFIX | Brazil |  |  |  |  |
|  |  | USDA 110 | *B. diazoefficiens* |  | USA |  |  |  |  |
|  | - | USDA 110 | *B. diazoefficiens* | Nodule max | USA |  | 45 | 0 | Adjei-Nsiah et al. 2018 |
|  | Shitaochi (GH/Ah/001/15) |  |  |  |  | 0 | 3 | 0 | Dzomeku et al. 2019 |

Supplementary Table 2. Grain yield (kg ha-^1^) of rhizobia non-inoculated (-Rhz) and inoculated (+Rhz) strains on soybean, cowpea, and groundnut plants, respectively in Ghana soil conditions. Minimum, mean, maximum values, and the sample size (n) are presented.

|  |  | -Rhz | +Rhz | -Rhz | +Rhz |
| --- | --- | --- | --- | --- | --- |
| Soybean |  | 532c | | USDA 110 | |
|  | Maximum | 2600 | 2558 | 2047 | 2428 |
|  | Mean | 719 | 872 | 1200 | 1391 |
|  | Minimum | 56 | 86 | 360 | 432 |
|  | Sample size (n) | 82 | 82 | 10 | 10 |
|  |  | Nitragen-S | | Rhizobium sp. | |
|  | Maximum |  |  | 1900 | 2250 |
|  | Mean | 2650 | 3120 | 1825 | 2125 |
|  | Minimum |  |  | 1900 | 2000 |
|  | Sample size (n) | 1 | 1 | 2 | 2 |
| Cowpea |  | BR 3267 | | BR 3262 | |
|  | Maximum | 1368 | 2041 | 1368 | 2232 |
|  | Mean | 915 | 1304 | 837 | 1227 |
|  | Minimum | 520 | 700 | 175 | 410 |
|  | Sample size (n) | 12 | 12 | 5 | 5 |
|  |  | BR 3299 | | KNUST 1002 | |
|  | Maximum | 1368 | 2153 | 1229 | 1563 |
|  | Mean | 1120 | 1818 | 683 | 1086 |
|  | Minimum | 891 | 1459 | 175 | 465 |
|  | Sample size (n) | 4 | 4 | 13 | 13 |
|  |  | KNUST 1006 | | KNUST 1003 | |
|  | Maximum | 1229 | 1720 |  |  |
|  | Mean | 683 | 997 | 1110 | 1190 |
|  | Minimum | 175 | 410 |  |  |
|  | Sample size (n) | 13 |  | 1 | 1 |
|  |  | NC 92 | |  | |
|  | Maximum |  |  |  | |
|  | Mean | 1110 | 1240 |  |  |
|  | Minimum |  |  |  |  |
|  | Sample size (n) |  |  |  |  |
| Groundnut |  | USDA 110 | | USDA 3456 | |
|  | Maximum | 1770 | 2377 | 1450 | 2000 |
|  | Mean | 1243 | 1385 | 1212 | 1452 |
|  | Minimum | 707 | 765 | 1085 | 1145 |
|  | Sample size (n) | 8 | 8 | 3 | 3 |
|  |  | BR 3267 |  | KNUST 1001 | |
|  | Maximum | 1510 | 2101 | 1490 | 1365 |
|  | Mean | 1161 | 1404 | 1067 | 1062 |
|  | Minimum | 440 | 815 | 707 | 757 |
|  | Sample size (n) | 14 | 14 | 6 | 6 |
|  |  | KNUST 1002 | | KNUST 1003 | |
|  | Maximum | 1665 | 2175 | 1490 | 1521 |
|  | Mean | 1021 | 1196 | 977 | 1124 |
|  | Minimum | 305 | 515 | 440 | 745 |
|  | Sample size (n) | 21 | 21 | 7 | 7 |
|  |  | KNUST 1006 | | KNUST 1031 | |
|  | Maximum | 1665 | 1935 | 1490 | 1705 |
|  | Mean | 1003 | 1340 | 1067 | 1157 |
|  | Minimum | 305 | 775 | 707 | 756 |
|  | Sample size (n) | 15 | 15 | 6 | 6 |
|  |  | KNUST 1032 |  | NC 92 | |
|  | Maximum | 1490 | 1459 |  |  |
|  | Mean | 1067 | 1036 | 440 | 1170 |
|  | Minimum | 707 | 757 |  |  |
|  | Sample size (n) | 6 | 6 | 1 | 1 |

Supplementary Figure 1. Geographical coordinates of the experimental sites extracted from the articles published and used in the present work. Purple bullets represent imported/introduced rhizobia inoculant, and yellow bullets represent native isolated rhizobia species embedded in the inoculant formulations.


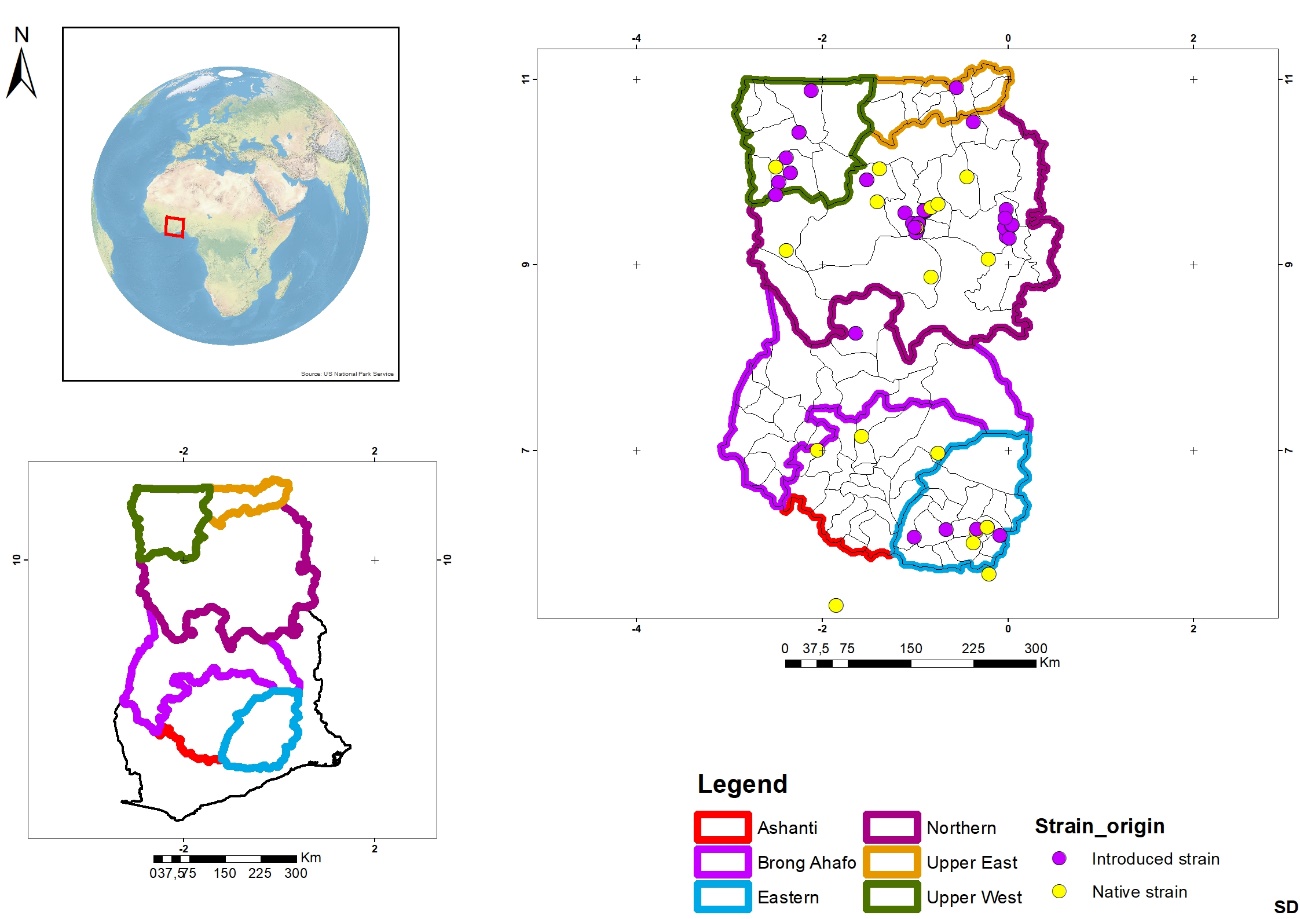


Papers used to construct the database

Adjei-Nsiah et al. 2018; Adjei-Nsiah et al. 2019; Adjei-Nsiah et al. 2022; Adusei et al. 2020; Ahiabor et al. 2014; Anani et al. 2021; Asante et al. 2020; Atakora et al. 2014; Avornyo et al. 2020; Awuni et al. 2020; Aziz et al. 2016; Boddey et al. 2016; Boddey et al. 2016; Dadson & Acquaah, 1984; Daramy et al. 2017; Dzomeku et al. 2019; Emmanuel et al. 2021; Emmanuel et al. 2021; Ezekiel-Adewoyin et al. 2017; Karikari et al. 2015; Atakora et al. 2014; Lambon et al. 2018; Masso et al. 2016; Miheretu & sarkodie-addo 2017; Miheretu & sarkodie-addo 2017;

Mintah et al. 2020; Mintah et al. 2020; Naabe et al. 2021a; Naabe et al. 2021b; Ofosu-budu et al. 2007; Osei et al. 2018; Osei et al. 2020; Ulzen et al. 2016; Ulzen et al. 2018; Ulzen et al. 2019; Ulzen et al. 2019; van Heerwaarden et al. 2018; Wilson et al. 2021;2021.
